# Advanced paternal age increases the risk of schizophrenia and obsessive–compulsive disorder in a Chinese Han population

**DOI:** 10.1016/j.psychres.2012.01.020

**Published:** 2012-08-15

**Authors:** Yuejing Wu, Xiang Liu, Hongrong Luo, Wei Deng, Gaofeng Zhao, Qiang Wang, Lan Zhang, Xiaohong Ma, Xiehe Liu, Robin A. Murray, David A. Collier, Tao Li

**Affiliations:** a The Mental Health Center and the Psychiatric Laboratory, West China Hospital, Sichuan University, Chengdu, Sichuan 610041, China; b State Key Laboratory of Biotherapy, West China Hospital, Sichuan University, Chengdu, Sichuan 610041, China; c Department of Psychological Medicine, Institute of Psychiatry, King's College London, London SE5 8AF, UK; d The MRC SGDP Centre, Institute of Psychiatry, King's College London, London SE5 8AF, UK

**Keywords:** Paternal age, Schizophrenia, Obsessive–compulsive disorder, Chinese population

## Abstract

Using the Structured Clinical Interview for DSM-IV, patient and non-patient version (SCID-P/NP), this study investigated 351 patients with schizophrenia, 122 with obsessive–compulsive disorder (OCD), and 238 unrelated healthy volunteers in a Chinese Han population. The relative risks posed by advanced paternal age for schizophrenia and OCD in offspring were computed under logistic regression analyses and adjusted for the participant's sex, age and co-parent age at birth. Compared to the offspring with paternal age of 25–29 years old, the relative risks rose from 2.660 to 10.183 in the paternal age range of 30–34 and ≥ 35. The relative risks for OCD increased from 2.225 to 5.413 in 30–34 and ≥ 35. For offspring with paternal age of < 25, the odds ratios of developing schizophrenia and OCD were 0.628 and 0.289 respectively, whereas an association between increased maternal age and risk for schizophrenia/OCD was not seen. Interaction analysis showed an interaction effect between paternal age and maternal age at birth. Such a tendency of risk affected by parental age for schizophrenia and OCD existed after splitting out the data of early onset patients. Sex-specific analyses found that the relative risks for schizophrenia with paternal age of 30–34 and ≥ 35 in male offspring were 2.407 and 10.893, and in female offspring were 3.080 and 9.659. The relative risks for OCD with paternal age of 30–34 and ≥ 35 in male offspring were 3.493 and 7.373, and in female offspring 2.005 and 4.404. The mean paternal age of schizophrenia/OCD patients born before the early 1980s was much greater than that of patients who were born after then. The findings illustrated that advanced paternal age is associated with increased risk for both schizophrenia and OCD in a Chinese Han population, prominently when paternal age is over 35. Biological and non-biological mechanisms may both be involved in the effects of advanced paternal age on schizophrenia and OCD.

## 1 Introduction

Neuropsychiatric disorders like schizophrenia usually are complex genetic diseases of unknown pathogenesis. There is a paradox that the worldwide incidence of schizophrenia appears to be largely stable, though it is highly heritable and confers a substantial reproductive disadvantage (McGrath et al., 1999; Haukka et al., 2003). Although schizophrenia is under strong negative genetic selection, its genetic risk alleles have not been gradually eliminated from the population (Keller and Miller, 2006; Uher, 2009). For decades, numerous studies have shown an association between advanced paternal age (APA) and increased risk for schizophrenia (Johanson 1958; Brown et al., 2002; Dalman and Allebeck 2002; Byrne et al., 2003; Zammit et al., 2003; El-Saadi et al., 2004; Sipos et al., 2004; Tsuchiya et al., 2005; Wohl and Gorwood 2007; Zammit et al., 2008). Furthermore, it was reported that “sporadic” patients with schizophrenia (i.e. without family history) are more likely to have older fathers than the familial patients (i.e. with family history) (Malaspina et al., 2002). Consequentially, APA is argued to be a crucial risk factor for schizophrenia.

Although mechanisms underlying the role of APA in adverse health outcomes in offspring are unclear, it is suggested that accumulated de novo mutations and/or aberrant epigenetic regulations in spermatogenesis with aging are critical (Crow 1999; Malaspina 2001; Perrin et al., 2007). The biological mechanisms of APA have also been discussed in studies of other neuropsychiatric disorders, such as autism (Reichenberg et al., 2006; Tsuchiya et al., 2008), bipolar disorder (Frans et al., 2008), and non-psychiatric diseases including nervous system cancers (Hemminki and Kyyronen, 1999), achondroplasia (Laxova, 1998), Marfan syndrome (Murdoch et al., 1972; Tarin et al., 1998), Apert syndrome (Glaser et al., 2003; Yoon et al., 2009), and even intelligence and neurocognitive performance (Malaspina et al., 2005; Saha et al., 2009).

Along with the biological hypotheses of the adverse role APA plays in schizophrenia, there is an alternative hypothesis that the selection of late fatherhood accompanies a predisposition to schizophrenia (Petersen et al., 2011). Since the early 1980s, great social transitions have taken place in China, such as urbanization, industrialization and improvements in education (Cai 2010), as well as attitudes toward marriage and parenthood (Zhenzhen et al., 2009). We wondered whether the time for childbearing changed along with social transitions and implementation of the one-child policy in China, and had an influence on the susceptibility to certain psychiatric disorders.

Until now, APA has rarely been studied in the anxiety disorders such as obsessive–compulsive disorder (OCD). Considering the common neurological soft signs observed in schizophrenia and the obsessive-compulsive spectrum disorders (Tumkaya et al., 2010), the high co-morbidity rates for OCD in schizophrenia (Lysaker et al., 2000; Bottas et al., 2005; Kayahan et al., 2005; Reznik et al., 2005), and the common pathways (e.g. related to dopaminergic and serotonic pathways) in schizophrenia and OCD (Tibbo and Warneke 1999; Azzam and Mathews 2003; Meira-Lima et al., 2004; Zinkstok et al., 2008), it has been argued that these two diseases may have some common predisposing factors in neurodevelopment (Tumkaya et al., 2010) and biological mechanisms.

The previous study of our group has replicated the finding of association between APA and increased risk for schizophrenia in a Chinese Han population (Wu et al., 2011). In the present study, we aimed 1) to investigate whether APA also increases the risk for OCD, and whether the risks attributable to APA for schizophrenia and OCD are similar or not, 2) to detect whether the risk from APA for schizophrenia/OCD affects male and female offspring differently, and 3) to explore whether the time of fatherhood and motherhood changed in the parents of participants along with the social transition.

## 2 Methods

### 2.1 Participants

The study was conducted in the Mental Health Center of West China Hospital, Sichuan University, as part of our series of genetic studies in a number of psychiatric disorders (Li et al., 2004; Zhang et al., 2004; Ma et al., 2007; Wang et al., 2007). Patients with schizophrenia and OCD were drawn from the inpatient and outpatient units of the Mental Health Center from January 2003 to December 2006. The patients were enrolled in this analysis if the information about sex, age and their parents' ages at birth was available. The patients were screened by trained psychiatrists with the Structured Clinical Interview for DSM-IV, Patient Version (SCID-P), which is based on the DSM-IV diagnostic criteria for mental disorders (First et al., 1997). The patients were excluded from this study if they 1) could not report either of their parents' ages at the time of their birth or 2) had co-morbid and obsessive–compulsive symptoms according to SCID-P.

The unrelated healthy controls were enrolled as volunteers who were willing to attend an anonymous survey about their demographic information and receive a current mental health assessment for free in the psychiatric department of West China Hospital. The first part of the assessment was a face-to-face interview with a trained psychiatrist, and the second part was a cognitive function test if the volunteers were willing to be evaluated. The exclusion criteria of control participants in the present study were as follows: 1) those who could not report either of their parents' ages at the time of their birth, 2) those who were diagnosed to have had psychiatric disorders or were suffering from psychiatric disorders according to the Structured Clinical Interview for DSM-IV, Non-Patient Version (SCID-NP) (First et al., 1997), and 3) those who had a family history of psychiatric disorders.

Written informed consent was obtained from each participant. This study was approved by the Ethics Committee of West China Hospital, Sichuan University.

### 2.2 Statistical analysis

#### 2.2.1 Demographic features of the participants

To estimate the heterogeneity among the three groups, a chi-square test was used to compare the sex distribution in the three adult groups by cross-tabulation; independent-samples *t* tests were used in the comparisons of participants' current age, and paternal and maternal age at birth, respectively.

#### 2.2.2 Logistic regression analyses

It is suggested that the 25–29 year old range is the most common reference category in the majority of previous studies about risk affected by paternal and maternal age for schizophrenia (Miller et al., 2011). Besides, the mean parental age in the healthy control group belonged to the 25–29 year old range in the present study. We set the 25–29 year old category as reference. Due to the small sample size of fathers aged below 20 or over 40 in both the patient groups and the control group, we set the lowest and highest paternal age category at < 25 and ≥ 35 years old. Paternal and maternal ages were divided into four categories: < 25, 25–29, 30–34, and ≥ 35.

The *P* values, odds ratios (ORs) and 95% Confidence Intervals (95% CIs) of each paternal age category were calculated by performing logistic regression analyses. These results were then adjusted for the participant's sex, current age and maternal age at birth accordingly. The same processes were utilized in the analysis of maternal age at birth as well. An interaction analysis for parental age at birth was chosen under the logistic regression model to explore whether there was an interaction effect between paternal and maternal age at birth, which could affect the risk for schizophrenia/OCD in offspring.

We stipulated the following: 1) etiological heterogeneity exists in the subtypes of schizophrenia and OCD with different ages of onset (early onset means having the first onset at or before 16, and the late onset means having the first onset after 16) (Taylor 2011), 2) APA may have a different impact on the risk for schizophrenia in male and female offspring (Miller et al., 2011). We stratified the participants by age-of-onset and sex, and repeated logistic regression analyses to compute the relative risk for schizophrenia and OCD in offspring.

#### 2.2.3 Time of parenthood before and after social transition in China

In addition to the report that the selection of delayed fatherhood may be related to the effect of APA for schizophrenia (Petersen et al., 2011), many social environmental factors are suggested to be essential in the etiology of schizophrenia (Brown 2011), for example, time trend, migration, and socio-economic status. Great socio-economic changes have taken place since the early 1980s in China (Cai, 2010). Contrary to the Chinese tradition of early marriage and large families, many young Chinese couples nowadays not only postpone marriage and childbearing, but also voluntarily eschew having more children in response to economic and social pressures, and in accord with their own personal career and life goals (Zhenzhen et al., 2009).

In the present study, we took the start of parenthood into account and set a cut-off of 25 years old in participants, which coincides with the beginning of the period of social transition in China. We performed comparisons by independent-samples *t* tests to explore whether the time for childbearing changed along with the social transition and varied in groups of schizophrenia patients, OCD patients, and healthy participants.

*P*-values were considered statistically significant at the α = 0.05 level. Statistical analyses were performed using the SPSS v15.0 software package.

## 3 Results

### 3.1 Demographic features

In total, 351 patients with schizophrenia (167 males, 134 females), 122 patients with OCD (67 males, 55 females), and 238 healthy controls (122 males, 116 females) were recruited in the present study. There were 35 patients with schizophrenia and 17 patients with OCD who had their onset at or before age 16. There were 364 participants who were born before the early 1980s (187 with schizophrenia, 50 with OCD and 127 healthy participants) and 347 born after (164 with schizophrenia, 72 with OCD and 111 healthy participants). The demographic characteristics of the three groups are shown in Table 1.

There were no differences in the sex distributions of the three groups (Pearson *χ*^2^ = 0.780, *P* = 0.677). The current age of patients with schizophrenia (25.80 ± 7.668) was slightly higher than that of healthy participants (24.26 ± 5.820) (*F* = 40.668, *t*′ = 2.764, *P* = 0.006), and there was no difference between the current age of participants with OCD (23.17 ± 6.984) and that of healthy participants. The paternal ages in both participants with schizophrenia and OCD were greater than those of healthy participants (*F* = 16.275, *t*′ = 6.196, *P* < 0.001, and *F* = 8.965, *t*′ = 4.961, *P* < 0.001, respectively). The maternal ages of participants with schizophrenia/OCD and that of healthy participants did not differ (See Table 1).

### 3.2 Logistic regression analyses for risk affected by paternal and maternal age

By using logistic regression models, setting the 25–29 category as reference, and adjusting for the participant's sex, age and maternal age at birth, the ORs for schizophrenia were 0.628 in < 25 (*P* = 0.119, 95% CI: 0.350–1.127), 2.660 in 30–34 (*P* < 0.001, 95% CI: 1.697–4.169), and 10.183 in ≥ 35 (*P* < 0.001, 95% CI: 4.772–21.729). The ORs for OCD were 0.289 in < 25 (*P* = 0.017, 95% CI: 0.105–0.800), 2.225 in 30–34 (*P* = 0.005, 95% CI: 1.266–3.909), and 5.413 in ≥ 35 (*P* < 0.001, 95% CI: 2.154–13.602).

Table 2 shows the ORs for schizophrenia and OCD related to maternal age as determined by logistic regression analyses. Except for the OR of mothers in the < 25 category for schizophrenia in offspring (*P* = 0.023, OR = 1.635, 95% CI: 1.069–2.500), the ORs for schizophrenia and OCD related to maternal age were all non-significant (*P* > 0.05)**.** Interaction analyses between paternal age and maternal age at birth were observed both in the group of schizophrenia (*P* < 0.001, OR = 1.002, 95% CI: 1.001–1.002) and OCD patients (*P* < 0.001, OR = 1.002, 95% CI: 1.001–1.003).

In short, the risk for schizophrenia/OCD increased when paternal age rose, whereas the risk of schizophrenia and OCD showed no statistically significant increase in relation to maternal age.

### 3.3 Specific analyses

#### 3.3.1 Age-of-onset

In the late onset subgroup, The ORs associated with paternal age for OCD were 0.516 in paternal age of < 25 (*P* = 0.052, 95% CI: 0.265–1.005), 3.188 in 30–34 (*P* < 0.001, 95% CI: 1.958–5.188), and 9.828 in ≥ 35 (*P* < 0.001, 95% CI: 4.538–21.286) after adjusting for the participant's sex, current age and maternal age at birth. The ORs effected by paternal age for OCD were 0.243 in < 25 (*P* = 0.028, 95% CI: 0.069–0.860), 2.652 in 30–34 (*P* = 0.002, 95% CI: 1.432–4.914), and 5.320 in ≥ 35 (*P* = 0.001, 95% CI: 2.067–13.716). As to the early onset subgroup, the ORs in paternal age categories (< 25, 30–34, and ≥ 35) for schizophrenia and OCD were all non-significant (*P* > 0.05)**.** See Table 2.

The results illustrated that the association of advanced paternal age with increased risk for schizophrenia/OCD existed even when we eliminated the data of the early onset participants. The non-significant ORs calculated in the early onset subgroup may be due to the relatively small sample (35 in the group of schizophrenia, 17 in the group of OCD).

#### 3.3.2 Sex of offspring

The ORs for schizophrenia in male offspring after adjustment were 0.565 in paternal age of < 25 (*P* = 0.200, 95% CI: 0.236–1.353), 5.407 in 30–34 (P = 0.004, 95% CI: 1.315–4.407), and 10.893 in ≥ 35 (*P* < 0.001, 95% CI: 3.754–31.609). The ORs for OCD in male offspring were 0.202 in paternal age of < 25 (*P* = 0.048, 95% CI: 0.041–0.987), 2.629 in 30–34 (*P* = 0.012, 95%CI: 1.242–5.564), and 6.819 in ≥ 35 (*P* = 0.003, 95% CI: 1.885–24.676). The ORs for schizophrenia in female offspring after adjustment were 0.695 in paternal age of < 25 (*P* = 0.369, 95% CI: 0.315–1.537), 3.082 in 30–34 (*P* = 0.001, 95% CI: 1.566–6.058), and 9.659 in ≥ 35 (*P* < 0.001, 95% CI: 3.249–28.715). The OR in paternal age of ≥ 35 category for OCD in female offspring was 4.029 (*P* < 0.039, 95% CI: 1.069–15.181). See Table 3.

The results indicated that the risk posed by advanced paternal age for schizophrenia was significantly increased both in male and female offspring, while the negative effect of advanced paternal age for OCD in female offspring could not be demonstrated.

### 3.4 Start of parenthood before and after social transition in China

Table 2 shows the mean parental age at birth, and the comparisons between the paternal and maternal age of participants born before and after the early 1980s in the three groups. The mean paternal ages of participants born before/after the social transition in both the group of patients with schizophrenia and the group of patients with OCD were significantly older than those of their healthy peers. The paternal age of participants with schizophrenia/OCD who were born before the social transition was significantly greater than that of participants with schizophrenia/OCD born after, while there was no difference between the mean paternal age of healthy participants born before or after the social transition. Moreover, there was no difference among the comparisons of the start of motherhood in the three groups before and after social transition.

The results indicated that although the start of fatherhood was delayed in fathers of schizophrenia/OCD patients in comparison with healthy participants, selective delayed fatherhood was not observed in fathers of patients with schizophrenia/OCD who were born after the early 1980s.

## 4 Discussion

### 4.1 Findings

Although some of the early studies reported inconsistent findings (Granville-Grossman, 1966), numerous studies have reported the association between APA and risk for schizophrenia (Johanson 1958; Brown et al., 2002; Dalman and Allebeck 2002; Byrne et al., 2003; Zammit et al., 2003; El-Saadi et al., 2004; Sipos et al., 2004; Tsuchiya et al., 2005; Wohl and Gorwood, 2007; Zammit et al., 2008). The present study found an association between APA and increased risk for both schizophrenia and OCD in a Chinese Han population, and that the risk for schizophrenia and OCD in offspring both grew in syncrony with APA. It was found that the later the beginning of fatherhood, the higher the risk of developing schizophrenia/OCD in the offspring. We refined the analyses of a previous study about APA and schizophrenia in a Chinese Han population (Wu et al. 2011) by stratifying the participants into early onset and late onset subgroups, and found that the risk associated with APA for schizophrenia/OCD existed even after splitting out the data of early onset participants with schizophrenia/OCD. There was no association between advanced maternal age at birth and increased risk for schizophrenia/OCD in offspring.

In the present study, we did not find a significantly different effect of APA on the risk for male and female offspring to develop schizophrenia/OCD. Previous studies reported inconsistent findings of the difference between the effect of APA for schizophrenia in male and female offspring. Byrne et al. (2003) conducted a case–control study based on a Danish longitudinal register, which recruited 7704 patients with schizophrenia and 192,590 age- and sex-matched controls with records of their family socioeconomic and demographic factors and family history. They found a gender effect that the risk conferred by advanced paternal age was particularly linked to female offspring who developed schizophrenia. Sipos et al. (2004) found no difference in the effect of advanced paternal age in the risk of schizophrenia for male and female offspring in a population-based cohort study. And in a meta-analysis, Miller et al. (2011) reported that younger paternal age (< 25) increased the risk for schizophrenia in male offspring but not female.

Comparing to the time to start fatherhood before the early 1980s, we did not observe selective delayed fatherhood after the early 1980s, while in the schizophrenia/OCD group, fathers of participants born after the early 1980s were much younger than those of participants who were born earlier. We attributed this finding partly to the fathers' usually having more than one child before the implementation of the family planning policy since the early 1980s, and partly to the influences of the social transitions that took place in China not only on attitudes toward marriage and childbearing. At the same time, there were also potential confounding risk factors for schizophrenia/OCD, for example, more expectations of parents for their only child's academic achievement, more stress for parents in the competition at the workplace, more problems in marriage and child–parent relationships, and less support for children in a nuclear family than in a large family formed by three generations (Xu, 2009).

#### 4.1.1 Explanations for the effect of paternal age in the biological aspect

Paternal age has been related to mutation in 1955 (Penrose, 1955). The earliest and classic studies which concerned the association between paternal age and schizophrenia (Johanson, 1958; Malaspina, 2001) explained that biological mechanisms underlie the risk posed by APA for schizophrenia. De novo mutations (DMNs) which occur during spermatogenesis as a consequence of fathers' aging and include de novo point mutation and de novo copy number variation. It is known that the much higher number of germ cell divisions in males, especially older males, results in higher mutation rate. Human female germ cells undergo 22 mitotic divisions before they enter the meiotic prophase (Drost and Lee, 1995), and then remain in meiotic arrest until adulthood, when ovulation takes place. In contrast, male germ cells divide continuously, with 23 mitotic divisions per year after puberty, resulting in 150 divisions by age 20 years and 840 replications by age 50 (Crow, 2000). There is also more vulnerability to temperature fluctuation in male germ cell division, and fewer DNA repair mechanisms available, meaning that male germ cells have more opportunities to accumulate mutations than females, especially in relation to male age (Risch et al., 1987).

The increased risk for individuals with older fathers of developing schizophrenia is also consistent with current hypotheses on the genetic architecture of complex genetic disorders. The architecture of schizophrenia is focused on its being composed of a mixture of both common and rare variants (Wray et al., 2010), although some propose that schizophrenia is caused exclusively by rare, even family-specific mutations which can only be detected by sequencing (McClellan and King, 2010). Recent empirical studies on the molecular genetic basis of schizophrenia have identified both common single nucleotide polymorphisms (SNPs) (International Schizophrenia Consortium et al., 2009; Stefansson et al., 2009) and rare, largely de novo, copy number variants (CNVs) (International Schizophrenia Consortium, 2008; Stefansson et al., 2008; Xu et al., 2008) associated with the disorder. In addition, rare non-synonymous variants have been identified in the ABCA13 gene (Knight et al., 2009) and de novo point mutations in synaptic genes such as SHANK3 (Gauthier et al., 2010). One current hypothesis is that at least a third of the genetic risk for schizophrenia derives from common variants of low risk (Raychaudhuri et al., 2010; Wray et al., 2010), 5–10% from rare CNVs (Need et al., 2009), and the remaineer being “dark matter” (Manolio et al., 2009) composed at least in part of rare and intermediate frequency variants, many of which may be de novo or of recent origin (Uher, 2009).

Although it might be expected that mutations are randomly spread throughout the genome, mutations in specific genes RET, FGFR2 and FGFR3 are exclusively paternal in origin and increase sharply with male age (Crow 2000). It has been reported that a rare structural variant that is associated with schizophrenia, such as the deletion of 22q11.2, is also related to OCD (Sebat et al., 2009). The finding of present study raises the issue of the biological effects of APA underlying the pathogenesis of schizophrenia and OCD.

The alternative biological mechanism involves the epigenetic regulation of the genome, i.e. genomic imprinting (Malaspina, 2001). When imprinted genes inherited from the father are expressed, those inherited from the mother are silenced, and vice versa. Genomic methylation is one of the mechanisms for imprinting. It begins in the male germline in the fetal gonad before birth, and is completed during postnatal spermatogenesis; it can be erased or re-established late in spermatogenesis (Zamudio et al., 2008) for paternally imprinted genes. The process of imprinting is vulnerable as paternal age advances. There are already some studies which show that imprinted genes play a key role in brain development (Keverne et al., 1996; Isles and Wilkinson, 2000), such as in Turner syndrome (Skuse et al., 1997), Angelman syndrome (Mann and Bautolomei, 1999), and autism (Schroer et al., 1998).

#### 4.1.2 Explanations for the effect of paternal age in a social and environmental aspect

Aside from the biological hypotheses to explain the effect of paternal age for adverse health outcome in offspring, alternative non-biological hypotheses include the selection of delayed fatherhood which may accompany a predisposition to schizophrenia (Petersen et al., 2011), the young fathers' lifestyle leading to birth defects in the offspring (Kazaura et al., 2004), the biological fathers' non-residence (which can be due to divorce or loss of the father), poor parenting, and adverse psychopathology for psychological or psychiatric problems in the offspring (Flouri, 2010). It is suggested that the quality of father–child interaction is significantly associated with emotional and behavioral outcomes in both young and older offspring even after controlling for the quality of mother–child interaction, while the mother–child interaction is stronger on emotional and behavioral outcomes only in young children (Flouri, 2010). Thus, the social and environmental context of fatherhood, which is partly related to the father's age at the child's birth and social and cultural trends, affects susceptibility to schizophrenia/OCD in the offspring (Brown 2011).

### 4.2 Strengths and weaknesses

In the present study, we use a “narrow” definition of schizophrenia rather than a broader definition of psychosis. Brown et al, (2002) demonstrated that the strength of the risk conferred by paternal age according to the diagnoses was higher for schizophrenia than for other psychoses (Brown et al., 2002). A similar argument was also seen in other studies (Malaspina, 2001; Zammit et al., 2003). Recently, in studies of other types of psychosis, such as bipolar disorder, the paternal age effect is less pronounced (Frans et al., 2008), and has not been associated with de novo copy number variation (Grozeva et al., 2010).

Some previous studies concerning the association between APA and schizophrenia had sensitive designs, such as being based on a birth register system and well-matched data of economic status of parents (Byrne et al., 2003), taking into account the family history of psychiatric disorders or psychosis-like symptoms (Zammit et al., 2008), and comparing the risk for schizophrenia in offspring with different birth orders (Petersen et al., 2011). Besides, there are many other confounding factors associated with schizophrenia, which have been discussed in former studies. These confounding factors include parental education, social class at birth (Corcoran et al., 2009), exposure to early traumatic experiences (Morgan and Fisher, 2007), like early loss of a parent (Agid et al., 1999), urbanicity (Pedersen and Mortensen, 2001a), ethnicity (Miller et al., 2011) and immigration (Cantor-Graae and Selten, 2005; Cooper, 2005), birth events (Clarke et al., 2006), seasons at birth and latitude in prenatal periods (McGrath and Welham, 1999; Pedersen and Mortensen, 2001b), etc. In the present case–control study, we could not add these crucial factors in analyses due to lack of data. Further studies are needed to confirm current findings of this study and to explore the genetic and non-genetic risk factors associated with paternal age, which require collaboration across different disciplines, including epidemiology and genome sequencing.

## Figures and Tables

**Table 1:** Demographic features of the participants.

Demographic features		Schizophrenia		OCD		Control	
		Male	Female	Male	Female	Male	Female
Paternal age categories	< 25	13	16	2	3	17	18
	25–29	69	60	27	26	65	63
	30–34	62	51	27	16	32	26
	≥ 35	48	31	11	10	8	9
Participant's age (*M* ± S.D. years)		25.80 ± 7.668		23.17 ± 6.984		24.26 ± 5.820	
Paternal age (*M* ± S.D. years)		30.55 ± 4.901		30.98 ± 5.301		28.29 ± 3.945	
Maternal age (*M* ± S.D. years)		26.94 ± 4.184		27.39 ± 4.677		26.51 ± 3.699	

**Table 2:** Risk related to paternal/maternal age for schizophrenia/OCD in male/female offspring.

After adjustment		P	OR	95%CI	*P*	OR	95%CI	P	OR	95%CI	P	OR	95%CI
		Risk affected by paternal age for SCZ			Risk affected by paternal age for OCD			Risk affected by maternal age for SCZ			Risk affected by maternal age for OCD		
	< 25	0.119	0.628	0.350–1.127	0.017	0.289	0.105–0.800	0.023	1.635	1.069–2.500	0.132	1.549	0.877–2.737
Parental age categories	25–29	1			1			1					
	30–34	< 0.001	2.660	1.697–4.169	0.005	2.225	1.266–3.909	0.221	0.717	0.421–1.222	0.294	0.698	0.357–1.366
	≥ 35	< 0.001	10.183	4.772–21.729	< 0.001	5.413	2.154–13.602	0.370	0.581	0.177–1.903	0.214	0.398	0.093–1.703
		Risk affected by paternal age for SCZ in male offspring			Risk affected by paternal age for OCD in male offspring			Risk affected by paternal age for SCZ in female offspring			Risk affected by paternal age for OCD in female offspring		
Paternal age categories	< 25	0.200	0.565	0.236–1.353	0.048	0.202	0.041–0.987	0.369	0.695	0.315–1.537	0.150	0.377	0.100–1.422
	25–29	1			1			1			1		
	30–34	0.004	2.407	1.315–4.407	0.012	2.629	1.242–5.564	0.001	3.080	1.566–6.058	0.226	1.710	0.717–4.076
	≥ 35	< 0.001	10.893	3.754–31.609	0.003	6.819	1.885–24.676	< 0.001	9.659	3.249–28.715	0.039	4.029	1.069–15.181

Abbreviations: SCZ = schizophrenia, OCD = obsessive–compulsive disorder, HC = healthy control. *P*, OR, 95% CI were adjusted for participant's sex, present age and co-parental age. There was one patient with schizophrenia who did not have the record of paternal age at birth, and three patients with schizophrenia and two healthy participants who did not have the record of maternal age at birth.

**Table 3:** Independent-samples *t* tests for the comparisons of paternal age before and after the 1980s.

*P* values	*M* ± S.D. years	SCZ^a^ (186)	OCD^a^ (50)	HC^a^(127)	SCZ^b^ (164)	OCD^b^ (72)
		31.34 ± 4.926	31.98 ± 6.482	28.28 ± 3.881		
SCZ^b^ (164)	29.66 ± 4.730	0.001				
OCD^b^ (72)	30.29 ± 4.207		0.048			
HC^b^ (111)	28.29 ± 4.035			0.994	0.016	0.004
SCZ^a^ (186)				< 0.001		
OCD^a^ (50)				< 0.001		

Abbreviations: SCZ = schizophrenia, OCD = obsessive–compulsive disorder, HC = healthy controls.

a Participants who were born before the early 1980s.

b Participants who were born after the early 1980s.
